# Genome Sequence of Porcine *Escherichia coli* Strain IMT8073, an Atypical Enteropathogenic *E*. *coli* Strain Isolated from a Piglet with Diarrhea

**DOI:** 10.1128/genomeA.00573-13

**Published:** 2013-08-01

**Authors:** Torsten Semmler, Inga Eichhorn, Astrid Bethe, Rolf Bauerfeind, Derek Pickard, Robert A. Kingsley, Gordon Dougan, Lothar H. Wieler

**Affiliations:** Institute of Microbiology and Epizootics, Freie Universität Berlin, Berlin, Germanya; Institute of Hygiene and Infectious Diseases of Animals, Justus Liebig University Giessen, Giessen, Germanyb; Pathogen Genomics, the Wellcome Trust Sanger Institute, Hinxton, Cambridge, United Kingdomc

## Abstract

*Escherichia coli* is a highly diverse bacterial species, with atypical enteropathogenic *E. coli* (aEPEC) causing intestinal disease in both human and animal hosts. Here, we report the first complete genome sequence of an aEPEC strain of sequence type ST794 and serotype Ont:H7, isolated from a diseased piglet.

## GENOME ANNOUNCEMENT

*Escherichia coli* is a Gram-negative bacterium of mammals and various bird species. *E. coli* bacteria are classified by the disease they cause and their genetic contents in commensal strains and specific pathogenic strains (1, 2). Both intestinal and extraintestinal pathogenic *E. coli* (InPEC and ExPEC) display distinct pathovars, some of which are known as zoonotic (1, 3, 4). Typical enteropathogenic *E. coli* (EPEC) has so far been detected only in humans (1). However, the closely related atypical EPEC (aEPEC) has been isolated from human and animal hosts, like cattle, swine, birds, and dogs, both in diseased and healthy status (3, 5–12). Thus, due to the etiological role of aEPEC bacteria in animal disease and their potential zoonotic relevance, animals could be a source for human infection (7, 13).

Here, to the best of our knowledge, we publish the first genome sequence of a porcine aEPEC strain. Strain IMT8073 was isolated in 2003 from a four-day-old suckling piglet suffering from diarrhea, although the sow had been vaccinated against enterotoxigenic *E. coli* (ETEC) during pregnancy. In addition to determining the genome sequence, which now enables a genomic comparison of human and porcine strains, we characterized IMT8073 using several phenotypical tests (data not shown).

IMT8073 reacted positively in the fluorescent actin staining (FAS) test in both human HEp-2 and porcine IPEC-J2 cells. A FAS-positive reaction resembles the attaching and effacing (A/E) lesion, which is associated with intestinal virulence in both human and animal hosts (3). The serotype Ont:H7 has previously been reported to be one of the rarer serotypes of aEPEC (11, 12).

DNA for genome sequencing was prepared from bacteria grown in Luria-Bertani (LB) medium, and sequencing was performed using a combination of Roche GS-FLX titanium and Illumina HiSeq 2000 sequencing. The use of 454 GS-FLX library preparation and sequencing resulted in the generation of 943,634 paired reads with an 8-kb insert, while by Illumina sequencing a total of 18,728,038 mate-pair reads were generated, with a 46-bp length on each side and a 3-kb insert between them. The 454 GS-FLX reads were assembled using Newbler version 2.8, which produced 18 scaffolds with sizes between 3 Mb and 2 kb. Thereafter, Illumina reads together with 454 GS-FLX reads were assembled with CLC Genomics Workbench version 6.0.3. The resulting 180 contigs (maximum length, 383 kb; minimum length, 200 bp; *N*_50_ contig size 177,556; mean coverage 170; summing to a total genome size of 5,149,783 bp) were then ordered according to the scaffolds from the 454 assembly by an in-house-developed pipeline that contains ABACAS (14).

Genomic features allowing the identification of aEPEC were demonstrated in the resulting sequences, including the presence of the locus of enterocyte effacement (LEE)-pathogenicity island, demonstrated by the presence of *eae* (encoding intimin theta), *map*, *tir*, and other genes carried on the LEE, and the absence of both EPEC adherence factor (EAF)-plasmid and *stx* genes (10, 15). In addition, several virulence-associated genes known to be associated with aEPEC pathogenesis in pigs, like *paa*, *ent*, or *lifA* (6, 7, 10, 16), are present in the investigated aEPEC strain.

The sequence type of IMT8073 was determined as ST794. At the time of our search (20 June 2013), only one strain, of human origin and defined as nonpathogenic, was listed in the database (http://mlst.ucc.ie/mlst/dbs/Ecoli).

### Nucleotide sequence accession numbers. 

This whole-genome shotgun project has been deposited at DDBJ/EMBL/GenBank under the accession number ASXQ00000000. The version described in this paper is version ASXQ01000000.
